# Supplementary material for: Aquibium pacificus sp. nov., a Novel Mixotrophic Bacterium from Bathypelagic Seawater in the Western Pacific Ocean
Source: Microorganisms. 2024 Aug 4;12(8):1584. doi: 10.3390/microorganisms12081584 (PMC11356281; doi:10.3390/microorganisms12081584)
Supplement: Supplementary file 1 [file microorganisms-12-01584-s001.zip › microorganisms-3129293-supplementary.pdf]

Supplementary Materials

Aquibium pacificus sp. nov., a novel mixotrophic bacterium from bathypelagic seawater in the western Pacific Ocean

Fan Jiang [1,2], Xun Hao [1], Ding Li [1,2], Xuying Zhu [1], Jiamei Huang [1], Qiliang Lai [1], Jianning Wang [1], Liping Wang [1,*] and Zongze Shao [1,*]

[1] Key Laboratory of Marine Genetic Resources, State Key Laboratory Breeding Base of Marine Genetic Resources, Fujian Key Laboratory of Marine Genetic Resources Third Institute of Oceanography, Ministry of Natural Resources of PR China, Xiamen, 361102, China

[2] College of Ocean and Earth Sciences, Xiamen University, Xiamen 361102, China

* Correspondence: liping84@tio.org.cn (L.W.); shaozongze@tio.org.cn (Z.S.)

Keywords: *Aquibium pacificus* LZ166^T; taxonomy; mixotrophy; carbon fixation; sulfur oxidation

Supplementary Materials

Figure S1. Transmission electron micrograph in exponential growth phase showing the cell morphology of strain LZ166^T.

Figure S2. Polar lipids of strain LZ166^T following separation by two-dimensional TLC.

Figure S3. Maximum likelihood phylogenetic tree based on 51 16S rRNA gene sequences showing the position between strain LZ166^T and other closely related phylogenetic neighbors.

Figure S4. Minimum evolution phylogenetic tree based on 51 16S rRNA gene sequences showing the position between strain LZ166^T and other closely related phylogenetic neighbors.

Figure S5. Neighbor joining phylogenetic tree based on 51 16S rRNA gene sequences showing the position between strain LZ166^T and other closely related phylogenetic neighbors.

Figure S6. Genes classification of strain LZ166^T against the RAST, COG and CAZy databases.

Figure S7. Phylogenetic analysis of form I and form II putative CoxL partial amino acid sequences based on alignments using ClustalW and analysis using MEGA7 with a neighbour joining algorithm.

Table S1. The Biolog GNIII test of strain LZ166^T.

Table S2. Cellular fatty acid compositions of strain LZ166^T and its reference strains.

Table S3. The average nucleotide identity(ANI), average amino identity(AAI) and digital DNA-DNA hybridization (dDDH) value (%) between strain LZ166^T and its close-related strains in *Aquibium*.

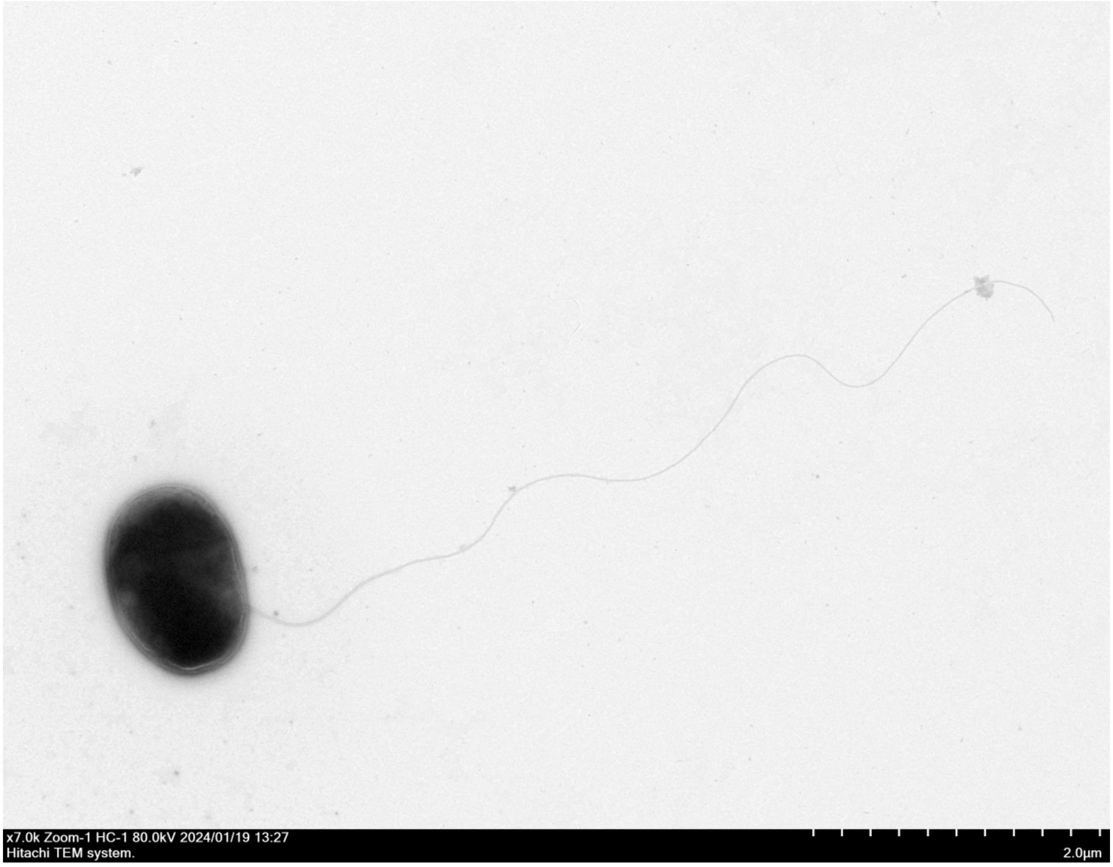

Figure S1. Transmission electron micrograph in exponential growth phase showing the cell morphology of strain LZ166[T]. Bar, 2.0μm.

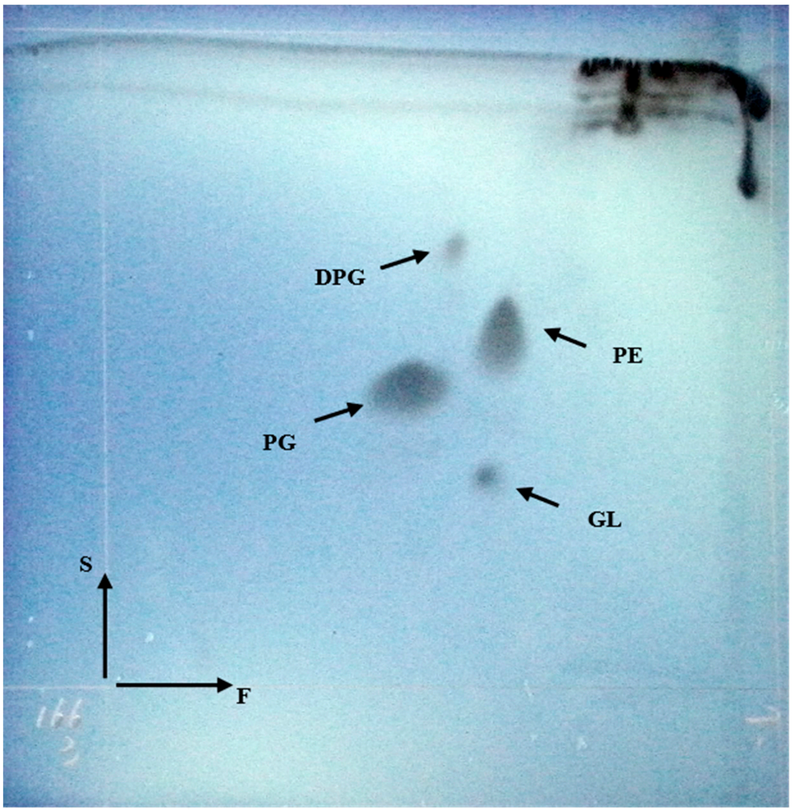

Figure S2. Polar lipids of strain LZ166^T following separation by two-dimensional TLC. DPG, diphosphatidylglycerol. PE, phosphatidylethanolamine. GL, glycolipid. PG, phosphatidylglycerol.

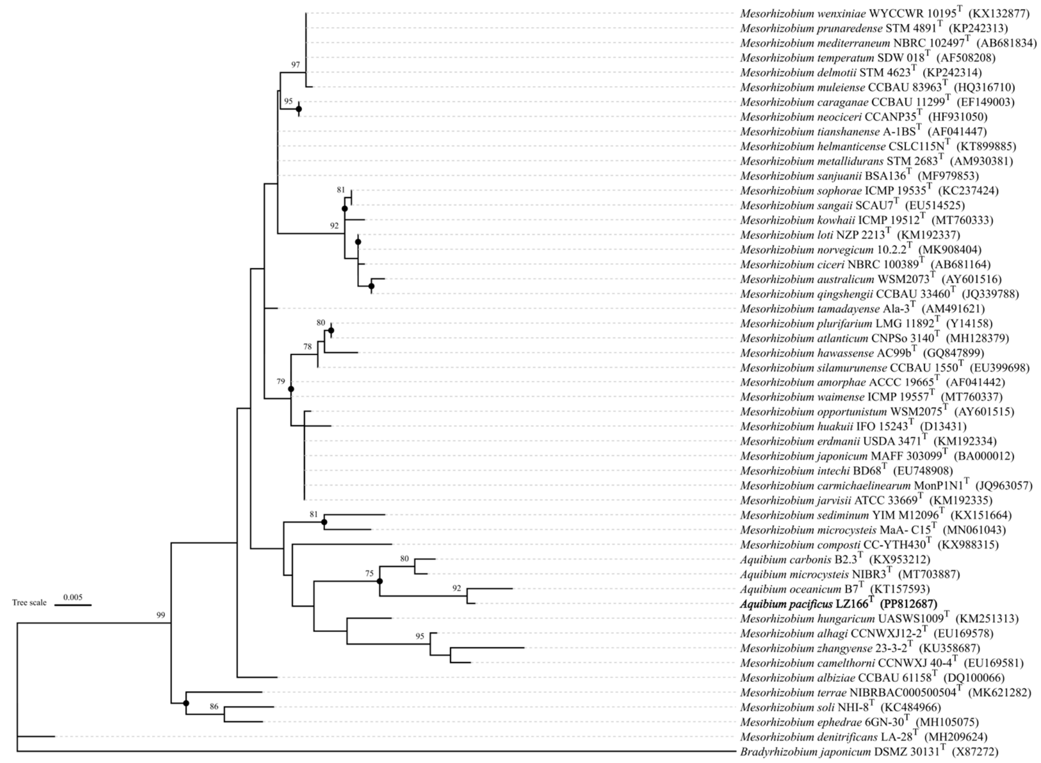

Figure S3. Maximum likelihood phylogenetic tree based on 51 16S rRNA gene sequences showing the position between strain LZ166^{T} and other closely related phylogenetic neighbours. Bootstrap numbers (>70%) were shown with 1000 calculations. Solid circle represented that branches were also recovered in the minimum evolution and neighbor joining trees. *Bradyrhizobium japonicum* DSMZ_30131^{T} (X87272) was used as the out group. Bar, 0.005 substitutions per nucleotide position.

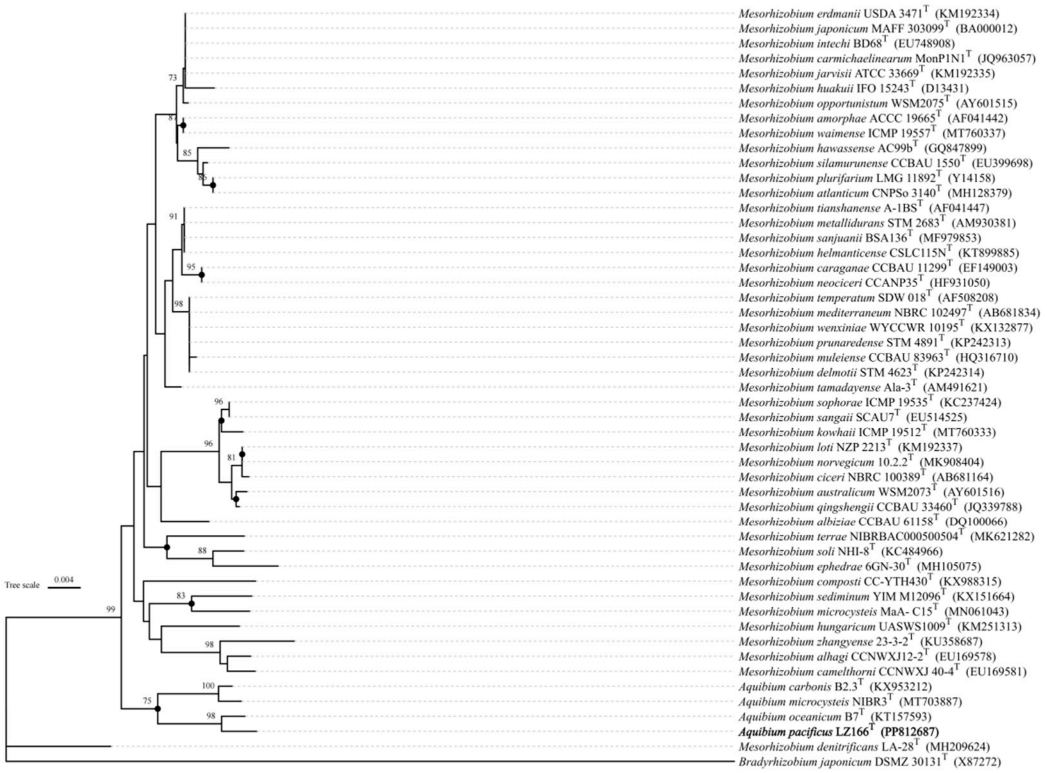

Figure S4. Minimum evolution phylogenetic tree based on 51 16S rRNA gene sequences showing the position between strain LZ166^T and other closely related phylogenetic neighbors. Bootstrap numbers (>70%) are shown with 1000 calculations. Solid circle represented that branches are also recovered in the maximum likelihood and neighbor joining trees. *Bradyrhizobium japonicum* DSMZ_30131^T (X87272) is used as the out group. Bar, 0.004 substitutions per nucleotide position.

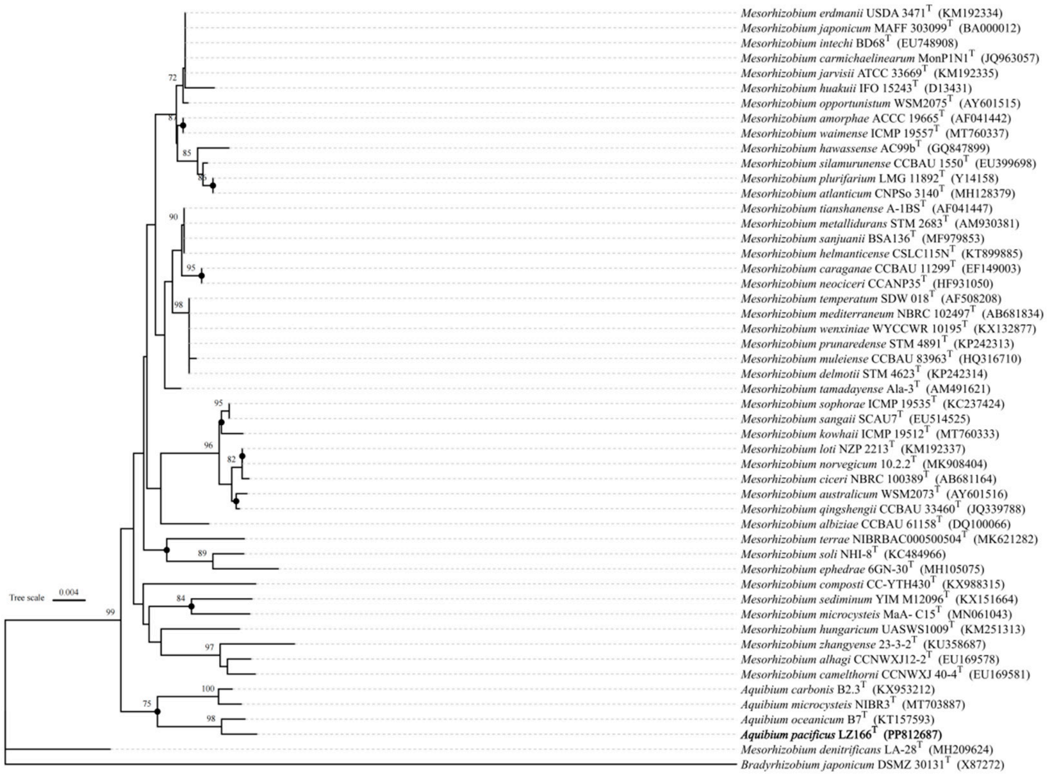

Figure S5. Neighbor joining phylogenetic tree based on 51 16S rRNA gene sequences showing the position between strain LZ166ᵀ and other closely related phylogenetic neighbours. Bootstrap numbers (>70%) are shown with 1000 calculations. Solid circle represented that branches are also recovered in the maximum likelihood and minimum evolution trees. *Bradyrhizobium japonicum* DSMZ_30131ᵀ (X87272) is used as the out group. Bar, 0.004 substitutions per nucleotide position.

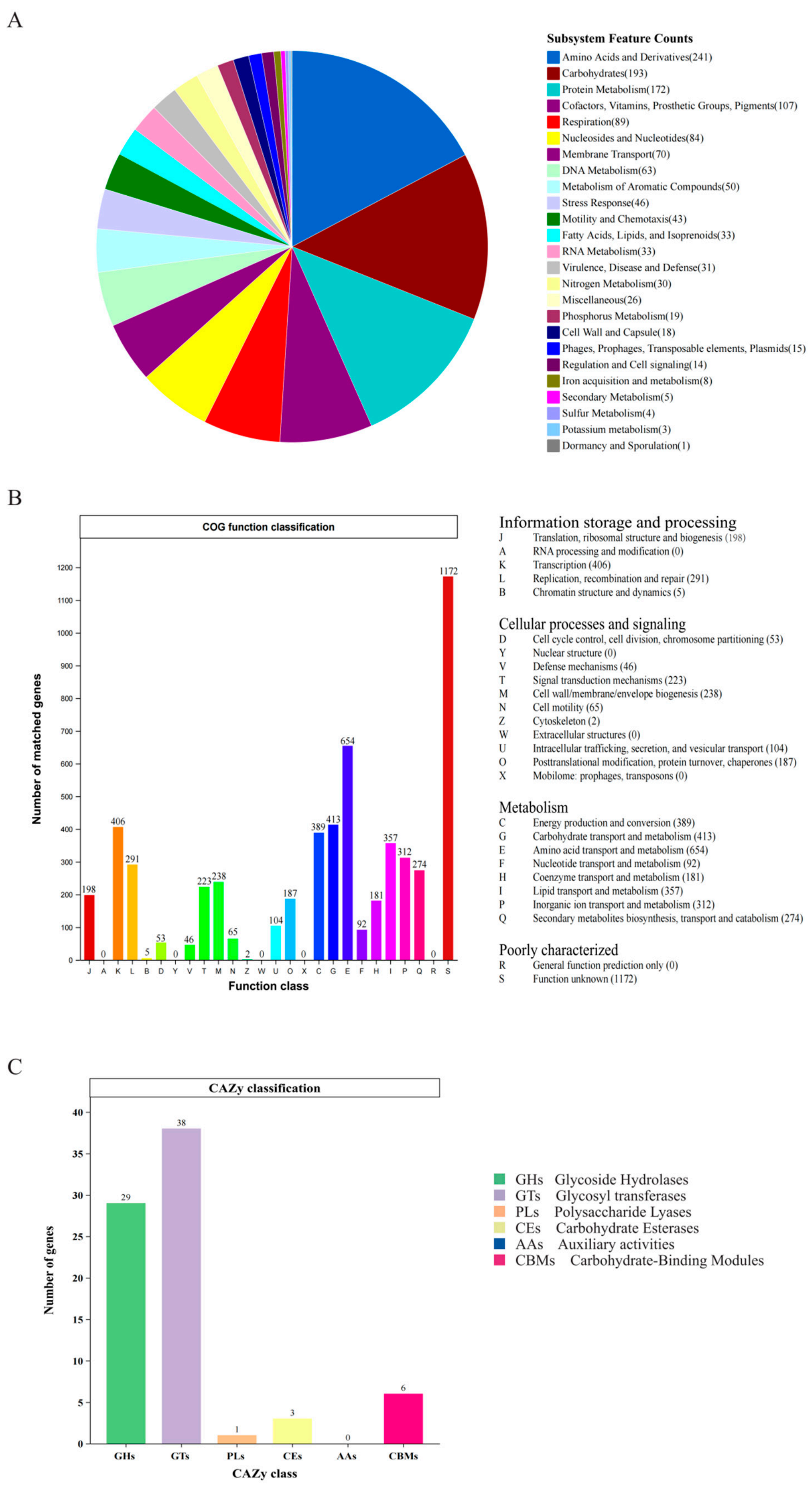

Figure S6. Genes classification of strain LZ166^T against the RAST, COG and CAZy databases. (A) the subsystem category number of genes by RAST annotation server. (B) number of genes associated with the 26 general COG functional categories. (C) Number of genes associated with the CAZy categories.

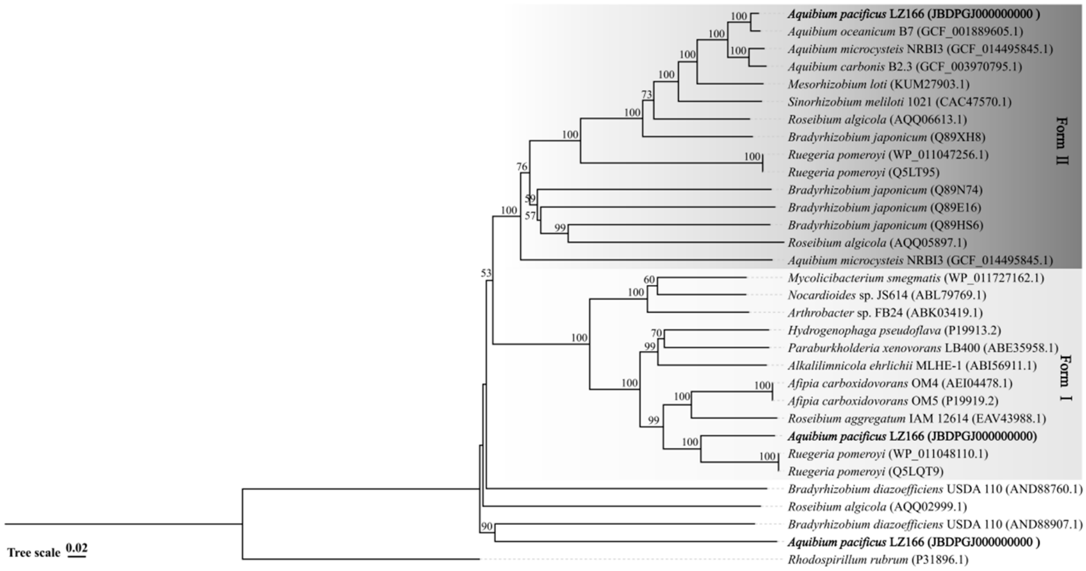

Figure S7. Phylogenetic analysis of form I and form II putative CoxL partial amino acid sequences based on alignments using ClustalW and analysis using MEGA7 with a neighbour-joining algorithm. Bootstrap values (%) are indicated at the branch nodes and were calculated from 1000-resample datasets. Threshold higher than 50% is displayed in the tree. Amino acid of Ni, Fe-CODH large subunit in *Rhodospirillum rubrum* (P31896.1) is used as the out group. Bar, 0.02 substitutions per amino position.

Table S1. The Biolog GNIII test of strain LZ166[T].

Biolog GNIII test		
Negative contract	inosine	**D-glucuronic acid**
**Dextrin**	1% sodium lactate	**Glucuronamide**
D-maltose	fusidic acid	Mucic acid
D-trehalose	D-serine	**Quinic acid**
**D-cellobiose**	**D-sorbitol**	D-saccharic acid
**Gentiobiose**	**D-mannitol**	Vancomycin
Sucrose	**D-arabitol**	**Tetrazolium violet**
**D-turanose**	Myo-inositol	Tetrazolium blue
Stachyose	Glycerol	p-hydroxyphenylacetic acid
**Positive contract**	D-glucose-6-PO_4	Methyl pyruvate
**pH6**	**D-fructose-6-PO_4**	D-lactic acid methyl ester
pH5	D-aspartic acid	L-lactic acid
D-raffinose	D-serine	Citric acid
α-D-lactose	Troleandomycin	**α-ketoglutaric acid**
D-melibiose	Rofamycin SV	D-malic acid
β-methyl-D-glucoside	Minocycline	L-malic acid
D-salicin	Gelatin	Bromosuccinic acid
N-acetyl-D-glucosamine	Glycyl-L-proline	**Nalidixic acid**
N-acetyl-β-D-mannosamine	L-alanine	Lithium chloride
N-acetyl-D-galactosamine	L-arginate	Potassium tellurite
N-acetyl-neuraminic acid	L-aspartic acid	Tween 40
**1%NaCl**	**L-glutamic acid**	γ-aminobutyric acid
4%NaCl	L-histidine	**α-hydroxybutyric acid**
8%NaCl	L-pyroglutamic acid	**β-hydroxy-D,L-butyric acid**
**α-D-glucose**	L-serine	α-ketobutyric acid
D-mannose	Lincomycin	**Acetoacetic acid**
**D-fructose**	Guanidine HCl	**Propionic acid**
D-galactose	Niaproof 4	**Acetic acid**
3-methyl glucose	Pectin	Formic acid
**D-fucose**	**D-galacturonic acid**	Aztreonam
L-fucose	**L-galactonic acid lactone**	**Sodium butyrate**
L-rhamnose	D-gluconic acid	Sodium bromate

Bold means positive.

Table S2. Cellular fatty acid compositions of strain LZ166^T and its reference strains.

Fatty acid (%)		**LZ166^T**	***A. microcysteis* NIBR3^T**	***A. oceanicum* B7^T**
Saturated	$C_{9:0}$	ND	0.2	ND
	$C_{11:0}$	0.2	0.1	ND
	$C_{16:0}$	**5.5**	3.6	**5.3**
	$C_{17:0}$	3.5	2.2	2.9
	$C_{18:0}$	4.1	**5.8**	**6.9**
	$C_{19:0}$	0.8	1.0	1.1
	$C_{20:0}$	1.6	2.4	1.4
Hydroxy	$C_{8:0}$ 3-OH	ND	0.1	ND
	$C_{10:0}$ 2-OH	ND	ND	0.7
	$C_{10:0}$ 3-OH	ND	ND	0.9
	$C_{11:0}$ 3-OH	ND	ND	0.3
	$C_{12:0}$ 3-OH	0.1	0.3	ND
	$C_{15:0}$ 2-OH	0.3	ND	ND
	$C_{16:0}$ 3-OH	0.4	ND	ND
	$C_{16:1}$ 2-OH	0.3	ND	ND
	$C_{18:0}$ 3-OH	0.5	0.1	0.4
Branched	iso-$C_{10:0}$	ND	ND	1.8
	iso-$C_{11:0}$	0.9	0.3	0.9
	iso-$C_{11:0}$ 3-OH	0.7	0.3	0.6
	iso-$C_{12:0}$ 3-OH	ND	ND	0.1
	iso-$C_{13:0}$ 3-OH	0.5	0.9	1.5
	iso-$C_{15:0}$	1.4	0.7	0.7
	iso-$C_{15:1}$ F	ND	0.4	1.1
	anteiso-$C_{15:1}$ A	ND	ND	0.4
	iso-$C_{16:0}$	ND	ND	0.2
	iso-$C_{17:0}$	**13.3**	4.3	**5.5**
	iso-$C_{17:0}$ 3-OH	0.2	ND	ND
	iso-$C_{19:0}$	0.8	0.7	0.4
	anteiso-$C_{19:0}$	ND	0.1	0.2
	$C_{19:0}$ cyclo $\omega 8c$	**9.3**	ND	**5.4**
Unsaturated	$C_{16:1}$ $\omega 11c$	ND	0.3	ND
	$C_{17:1}$ $\omega 6c$	0.7	0.5	0.5
	$C_{17:1}$ $\omega 8c$	1.0	1.7	0.9
	$C_{18:1}$ $\omega 9c$	1.1	1.4	2.4
	11-methyl $C_{18:1}$ $\omega 7c$	**12.0**	**11.6**	**9.6**
	$C_{20:1}$ $\omega 7c$	0.5	0.4	1.3
	$C_{20:2}$ $\omega 6,9c$	ND	ND	0.3
Summed feature	1*	ND	0.3	0.8
	2*	0.5	ND	ND
	3*	0.8	1.0	0.8
	4*	ND	0.2	0.4
	7*	ND	0.3	ND
	8*	**39.3**	**58.4**	**44.3**
	9*	0.36	0.3	ND

1* contains iso-$C_{15:1}$ H, $C_{13:0}$ 3-OH and/or i-$C_{15:1}$ H. 2* contains aldehyde -$C_{12:0}$ and/or unknown 10.928. 3* contains $C_{16:1}$ $\omega 7c$ and/or $C_{16:1}$ $\omega 6c$. 4* contains iso-$C_{17:1}$ I and/or anteiso-$C_{17:1}$ B. 7* contains unknown 18.846 and/or $C_{19:1}$ $\omega 6c$. 8* contains $C_{18:1}$ $\omega 7c$ and/or $C_{18:1}$ $\omega 6c$. 9* contains iso-$C_{17:1}$ $\omega 9c$ and/or 10-methyl $C_{16:0}$. Major fatty acid components (>5.0%) are showed in bold. ND, no detected. All data was obtained in this study.

Table S3. The average nucleotide identity(ANI), average amino identity(AAI) and digital DNA-DNA hybridization (dDDH) value (%) between strain LZ166^T and its close-related strains in *Aquibium*.

	A. oceanicum B7^T	*A. microcysteis* NIBR3^T	*A. carbonis* B2.3^T
ANI (%)	90.73	77.23	76.79
AAI(%)	88.50	79.71	79.03
dDDH(%)	36.1	22.8	22.2
